# Polish school nurses’ knowledge of the first-aid in tooth avulsion of permanent teeth

**DOI:** 10.1186/s12903-016-0183-2

**Published:** 2016-03-09

**Authors:** Joanna Baginska, Ewa Rodakowska, Robert Milewski, Magdalena Wilczynska-Borawska, Anna Kierklo

**Affiliations:** Department of Dentistry Propaedeutics, Medical University of Bialystok, Bialystok, Poland; Department of Restorative Dentistry, Medical University of Bialystok, Bialystok, Poland; Department of Statistics and Medical Informatics, Medical University of Bialystok, Bialystok, Poland; Department of Social and Preventive Dentistry, Medical University of Bialystok, Bialystok, Poland

**Keywords:** Tooth avulsion, School nurse, Knowledge, Self-education

## Abstract

**Background:**

The frequency of dental trauma in schools is secondary only to accidents at home. The aim of this study was to evaluate the knowledge of first aid in the avulsion of permanent teeth presented by Polish school nurses from different areas.

**Methods:**

A cross-sectional study with the use of a structured self-administrative questionnaire was conducted in 2014 on school nurses working in randomly selected Polish provinces. The instrument consisted of demographic questions, questions referring to nurses’ experience and training in dental trauma and questions checking knowledge of first-aid in the avulsion of permanent teeth. The maximum number of points to be scored was eight. Data were analyzed with the Kruskal-Wallis, the Mann–Whitney U and Chi^2^ tests with the level of statistical significance at *p* < 0.05.

**Results:**

The final sample consisted of 164 nurses of which 70.1 % had experience with dental injuries and 45.7 % witnessed a tooth avulsion in pupils. 10.4 % nurses participated in training courses concerning tooth avulsion and 67.1 % of them independently broadened their knowledge. The knowledge of the first-aid management of an avulsed tooth was moderate (4.72 ± 1.95 points). 78.1 % of nurses chose a correct definition of the term of ‘tooth avulsion’. Only 7.3 % of them were aware that the replantation could be conducted by any witness of an accident. Saline was most often chosen as a proper transport medium for an avulsed tooth (57.9 %), whereas 16.1 % of nurses indicated milk. 13.4 % of evaluated nurses showed readiness to conduct an immediate replantation. Most respondents preferred calling child’s parents and advising them to bring the child to a dentist (63.4 %). The main factor influencing nurses’ level of knowledge was self-education (*p* < 0.001). Being a witness to dental trauma (*p* = 0.0032) and working in schools with sports classes (*p* = 0.0423) were positive determinants of improved knowledge. Nurses from large agglomerations had significantly lower knowledge (*p* = 0.005). The main source of information for self-education was the Internet.

**Conclusions:**

The evaluated nurses were in need of education with regard to the management of dental trauma cases. The Internet should be used to deliver evidence-based knowledge to medical staff working at schools.

## Background

Traumatic dental injuries (TDI) are very common in school-age children. Glendor [1] estimated that almost a quarter of schoolchildren suffered from TDI, but the prevalence of dental trauma varied from country to country as well as within particular countries. The most common reasons for TDI are falls, sport activities, collisions and fights [2] which obviously may happen in or around school. The frequency of dental injuries in school takes the second place after accidents at home [3]. Rajab [4] reported that 25.6 % of TDI treated at the Department of Paediatric Dentistry of the University of Jordan between 1997 and 2000 happened at school.

Among several kinds of TDI, special attention should be paid to avulsion injuries, i.e. a complete displacement of a tooth from its socket in the alveolar bone, because the chances for preservation of the tooth are highly dependent on proper action taken at the site of accident. For the permanent dentition, the most desirable action is an immediate replantation of an avulsed tooth into its socket, which may be performed by anybody, even the victim, during first 5 min following the accident. An avulsed tooth should not be stored dry for a long time because after 30–60 min the majority of periodontal ligament cells will become necrotic, which significantly reduces the prognosis of periodontal healing. If immediate replantation is not possible, to prevent dehydration, the tooth should be stored in a transport medium that is a physiological solution which closely replicates the oral environment, especially its pH and osmolality. The most suitable media, like the Hank’s Balanced Salt Solution (HBSS), are not readily available to the general public, therefore milk, coconut milk, soya milk, normal saline and even patient’s saliva may be used as a short-time storage medium needed to transport the avulsed tooth to a dentist. There are also general rules that have to be followed in case of dental avulsion to prevent crushing of periodontal ligament cells: (i) the tooth should be held by the crown to avoid the damage of periodontal ligament on the root surface, (ii) a dirty tooth should be gently rinsed with water or saline, disinfectants and scrubbing of the root are inadvisable, (iii) the victim should be referred to a dentist as soon as possible [5–8].

In many cases the child’s accident would result in damages paid to injured children and their families by the founding bodies of educational institutions. The amount of such compensation may depend on the way in which first aid at the scene of the accident has been administered [9]. Undoubtedly school staff have to be prepared to provide emergency care of a pupil with dental injury. Such expectations are particularly addressed to nurses working at schools. In Poland, the Regulation of the Health Minister of 22 December 2004 on the Range and the Organisation of Preventive Healthcare of Children and Adolescents [10] imposes on school nurses, among other tasks, the obligation to administer premedical aid in case of emergency illnesses, injuries and poisoning. Polish school nurses’ preventive healthcare and first aid offices are located in schools. Nurses can practice as one-person offices or nurse partnerships, or be employed by a physician to provide preventive healthcare to the pupils. The average recommended number of pupils per one school nurse is between 30 and 1100 depending on the type of school and the degree of disability of the pupils. In most schools, the nurse is not present during the entire period when classes are held, but only at scheduled days and hours.

Only a few studies assessing school nurses’ knowledge and attitudes of first aid of tooth injuries were conducted. They revealed the necessity of including dental trauma in the nurses’ education [11, 12]. There is only one paper from Eastern European countries raising this problem [13]. However, all studies were conducted in large cities and so far the differences between nurses working in rural and urban areas have not been assessed. The aim of this study was to evaluate, by means of a structured self-administrative questionnaire, the knowledge of first aid in the avulsion of permanent teeth presented by Polish school nurses from different areas.

## Methods

### Study population

A descriptive cross-sectional study was conducted in the period from January to April 2014 under approval of the Bioethical Committee of the Medical University of Bialystok. Due to organizational reasons we chose a half of 16 Polish provinces using the Rand function from Microsoft Office Excel 2010. Then we searched the online database of the Central Statistical Office of Poland [14] for all nurse offices which stated that they provided healthcare in the education environment in the area of the drawn provinces. We found a total of 106 entities in the form of one-person nurse offices or nurse partnerships. The number of school nurses working in nurse partnerships or group offices was established by phone and all nurses from such entities were invited to participate. The exclusion criterion was not having a registered school nurse. Finally, 333 questionnaires with the information about the purpose of the study and a form for consent to the participation in the survey were sent. The letters were provided with return envelopes with prepaid postage.

### Instrument

A structured self-administrative questionnaire adopted in this survey had been previously used in the study of Baginska and Wilczynska [13]. Nurses received written information that the purpose of the survey was to assess the knowledge of avulsion in permanent dentition. The first part of the questionnaire consisted of 7 demographic questions regarding the time of work experience as a nurse and as a school nurse, the education level, the kind of location they worked in, the number of schools and children under care and the presence of a class specializing in sports in these schools. The second part of the questionnaire was divided into two sections: section I - 6 questions referring to the nurses’ experience and training in dental trauma and section II - 7 questions checking their knowledge of first-aid in dental avulsion.

To assess nurses’ knowledge, the answers to the questions from section II were scored with points. Questions referring to: a definition of tooth replantation, a person who should conduct this procedure, the best time for tooth replantation and the way of holding and cleaning an avulsed tooth had a key answer and could be scored with 1 or 0 point. The only multiple-response question concerned the storage of the tooth until replantation. One point was given exclusively for an answer containing at least one recommended medium (milk, physiological salt, patient’s saliva) without the indication of improper ways of storage of an avulsed tooth (sterile gauze, hand, water, alcohol, ice). If a surveyed nurse indicated even one improper way of tooth transport she received 0 points for that question. The possible score for the question “What would be your action in case of the necessity to provide first aid to a child with dental avulsion?” varied from 0 to 2 points. We gave 2 points for the answer: “immediate placement of a tooth into its socket” as it was the most proper behaviour, and 1 point was always given if the respondent chose the answer “call a dentist and follow her/his instructions”. In case of the answer “transport of the child to the nearest dentist” or “calling his/her parents with the instruction to transport of the child to the nearest dentist” the score was dependent on the answer to the question concerning the storage medium, i.e. 1 point if the nurse knew the proper storage medium and 0 points if an incorrect medium was indicated. The maximum number of points to be scored by any respondent was eight.

### Statistical analysis

The Statistica 10.0 software (StatSoft, Poland) was used for the calculations. Descriptive statistics were used to describe the study population. Means, median (Me), lower and upper quartile (Q1, Q3) of number of points scored by nurses were calculated with regard to their education level, location and type of class in school, having experience in dental trauma, training courses and self-education in dental trauma. The normality of distribution was verified using the Kolmogorov-Smirnov test with the Lilliefors correction and the Shapiro-Wilk test. The normality of distribution of analysed quantitative variables was not found. On this basis, non-parametric test for hypothesis testing were used. Kruskal-Wallis, the Spearman’s rank correlation coefficient and the Mann–Whitney U tests were used to assess factors influencing the level of knowledge of respondents. The Chi^2^ test was used to determine variables connected with the participation in training courses, and the self-education in the surveyed population. The level of statistical significance was established at *p* < 0.05.

## Results

One-hundred and sixty-four questionnaires with data suitable for statistical analysis were returned. Another returned questionnaire with only the demographic part filled in was not included in the analysis. The overall response rate was 49.25 %. Based on the following assumptions: 333 school nurses invited to the study, 70 % of them having experience with dental trauma, the maximum error of 5 % and the confidence level of 95 %, we established the minimum number of respondents needed for statistical analysis to be exactly 164 subjects.

The demographic background of the surveyed population is shown in Table 1. Our participants practiced the nursing profession between 8 and 47 years (mean 32 ± 7.57) and have been working as school nurses between 1 and 43 years (mean 23 ± 9.11). The vast majority of respondents (119 individuals, 72.6 %) reported to have the secondary education, whereas 20 (12.2 %) nurses graduated from an institution of higher education with a Bachelor’s degree, and 19 (11.6 %) individuals with a Master's degree. One hundred-fifty seven nurses answered the question about their location. 70 of them (42.7 %) worked in towns under 100000 of inhabitants, 28 (17.1 %) in towns under 20000 of inhabitants, 14 (8.5 %) nurses worked at village schools and 45 (27.4 %) in urban agglomeration areas (with the number of inhabitants over 100000). The number of pupils being under the care of the surveyed nurses was between 69 and 2132, 791 pupils on average. There were no differences in the number of pupils between nurses working in rural and urban areas. Only 64 (39 %) nurses worked at schools having classes specializing in sports.

**Table 1:** Sample characteristics

Demographic information	n	%
Education level		
Secondary	119	72.6
Bachelor’s degree	20	12.2
Master’s degree	19	11.6
No answer	6	3.6
Location		
Village	14	8.5
Town up to 20 000	28	17.1
City up to 100 000	70	42.7
City over 100 000	45	27.4
No answer	7	4.3
Sport class		
Yes	64	39
No	100	61

Table 2 depicts the experience of surveyed nurses in the field of dental trauma. In the entire population, 75 (45.7 %) subjects revealed that in the course of work as a school nurse they witnessed a tooth avulsion. Fights, misadventures and sport activities were indicated among the reasons for tooth avulsion in pupils. Moreover, 100 (60.9 %) subjects indicated that they witnessed dental trauma in situations outside of work. In total, 115 (70.1 %) nurses had experience with a dental injury. Only 17 (10.4 %) nurses participated in training courses concerning tooth avulsion, whereas as many as 110 (67.1 %) of them independently broadened their knowledge of this subject. Such individuals consulted a dentist (44.5 %), read the specialist literature (3 %), the press (27.3 %) and the online sources (65.5 %), and consulted other staff members (23.6 %).

**Table 2:** Experience and education in dental trauma

	n	%
Being a witness of tooth avulsion at work		
Yes	75	45.7
No	89	54.3
Being a witness of dental injury outside of work		
Yes	100	60.9
No	64	39.1
Having experience in dental injury		
Yes	115	70.1
No	49	29.9
Training in dental trauma		
Yes	17	10.4
No	147	89.6
Self-education		
Yes	110	67.1
No	54	32.9

Table 3 presents the answers to questions regarding the first-aid management of dental avulsion given by surveyed school nurses. In general, evaluated nurses understood the term of ‘tooth avulsion’, 128 (78.1 %) of them gave a correct answer. But only 12 (7.3 %) respondents were aware that the replantation of an avulsed tooth could be conducted by any witness of an accident. The knowledge that time was crucial for replantation and that an avulsed tooth needed to be properly held was also at a good level. Saline was most often chosen as a proper transport medium for an avulsed tooth, 95 (57.9 %) of respondents would use it. As much as one fifth of school nurses (20.1 %) chose the sterile gauze which was highly inadvisable. Only 22 (13.4 %) of evaluated nurses showed readiness to conduct an immediate replantation in case of a dental avulsion in their pupils. Other respondents would rather call the child’s parents and advise them to bring the child to a dentist (104, 63.4 %) or contact any dentist for advice (29, 17.7 %). The overall knowledge score obtained was 4.72 ± 1.95. The surveyed population included as many as 19 individuals who did not give any correct answer or scored only 1 point, whereas 6 nurses received the maximum score.

**Table 3:** Participants’ answers to questions regarding the knowledge of tooth avulsion

Question	n	%
Give the correct definition for replantation.		
Replacing avulsed teeth by other teeth	7	4.3
Placing back the same teeth which were avulsed	128	78.1
Placing an implant or denture in place of avulsed teeth	5	3.0
I don’t know	18	11
No answer	6	3.6
Who is the right person to conduct replantation?		
Only a dentist	140	85.4
Every person with medical background	3	1.8
Everybody who witnessed tooth avulsion	12	7.3
I don’t know	9	5.5
What time from trauma is crucial for the replantation?		
Up to 1 h	103	62.8
12 h	26	15.8
I don’t know	27	16.5
No answer	8	4.9
Avulsed teeth should be held by		
The crown	121	73.8
The root	0	0
I don’t know	29	17.7
No answer	14	8.5
Which medium is the best for the transport of an avulsed tooth?^a^		
Sterile gauze	33	20.1
Hand	1	0.6
Milk	27	16.5
Patient’s saliva	56	34.1
Saline	95	57.9
Water	12	7.3
Alcohol	0	0
Ice	12	7.3
What should be done if the avulsed tooth gets dirty?		
Wash it with water	138	84.1
Wash it using a detergent	7	4.3
Throw it away because if dirty it is useless	1	0.6
No answer	18	11
What would be your behaviour in case of tooth avulsion?		
Immediate provision of tooth replantation	22	13.4
Calling a dentist and following his/her instructions	29	17.7
Transporting the patient to the nearest dentist	6	3.7
Calling patient’s parents and advising them to take the child to a dentist	104	63.4
Keeping the child under observation	0	0
No answer	3	1.8

^a^ multiple-response question

Table 4 presents the level of knowledge of surveyed nurses depending on education, location, care for sports classes, experience in dental trauma and completion of post-graduate training courses on dental trauma. A statistical analysis showed that the factor which most favourably influenced the provision of correct answers was self-education. Nurses who declared that they had independently searched for information on the procedures in case of tooth avulsion obtained 5.17 points on average, and those who had not broadened their knowledge only 3.87 (*p* < 0.001, Mann–Whitney U test). Statistical analysis also showed that the knowledge of school nurses depended on their place of work. The individuals working in large agglomerations (over 100 000 inhabitants) scored fewer points than the nurses from towns and villages (*p* = 0.005, Kruskal-Wallis test). Being a witness to dental trauma (*p* = 0.032, Mann–Whitney U test) and the work in schools with sports classes (*p* = 0.0423, Mann–Whitney U test) also positively influenced nurses’ knowledge. The individuals who encountered a dental trauma showed interest in training courses and self-education (Table 5). There was no relationship between the number of children under their care and the level of school nurses’ knowledge (*p* = 0.2, Spearman’s rank correlation).

**Table 4:** The knowledge of surveyed nurses according to different variables (Q1 – lower quartile, Me- median, Q3 – upper quartile)

	Number of points				p
	mean	Q1	Me	Q3	
Education level					
Secondary	4.65	4	5	6	
Bachelor’s degree	5	4	5	6	0.81^a^
Master’s degree	4.89	3	6	6	
Location					
Village	4.93	5	6	6	
Town up to 20 000	5.36	4.5	6	6	**0.005** ^**a**^
City up to 100 000	4.98	4	6	6	
City over 100 000	3.75	2	4	6	
Sports class					
Yes	5.06	5	6	6	**0.04** ^**b**^
No	4.5	3.5	5	6	
Having experience in dental injury					
Yes	4.92	4	6	6	**0.03** ^**b**^
No	4.24	3	5	6	
Training in dental trauma					
Yes	5.29	5	6	6	0.23^b^
No	4.68	4	5	6	
Self-education					
Yes	5.17	4	6	6	**0.001** ^**b**^
No	3.87	2	5	6	

^a^Kruskal-Wallis test, ^b^Mann-Whitney U test. The bold data are statistically significant

**Table 5:** Participation in training courses on first aid in dental trauma and obtaining such knowledge by self-education according to different variables

	Training in dental trauma			Self-education		
	Yes N(%)	No N(%)	p^a^	Yes N(%)	No N(%)	p^a^
Education level						
Secondary	11 (9.3 %)	107 (90.7 %)		80 (67.8 %)	38 (32.2 %)	
Bachelor’s degree	3 (15 %)	17 (85 %)	0.73	14 (70 %)	6 (30 %)	0.98
Master’s degree	2 (10.5 %)	17 (89.5 %)		13 (68.4 %)	6 (31.6 %)	
Location						
Village	2 (14.3 %)	12 (85.7 %)		10 (71.4 %)	4 (28.6 %)	
Town up to 20 000	1 (3.5 %)	27 (96.5 %)	0.53	24 (85.7 %)	4 (14.3 %)	**0.03**
City up to 100 000	9 (12.8 %)	61 (87.2 %)		46 (65.7 %)	24 (34.3 %)	
City over 100 000	4 (9.1 %)	40 (90.9 %)		23 (52.3 %)	21 (47.7 %)	
Sports class						
Yes	5 (4.9 %)	58 (95.1 %)	0.40	44 (69.8 %)	19 (30.2 %)	0.61
No	88 (88 %)	12 (12 %)		66 (66 %)	34 (43 %)	
Being a witness of tooth avulsion at work						
Yes	14 (18.9 %)	60 (81.1 %)	**0.001**	57 (51.8 %)	53 (48.2 %)	**0.01**
No	3 (3.4 %)	86 (96.6 %)		17 (32.1 %)	36 (67.9 %)	
Being a witness of dental injury outside of work						
Yes	15 (15.2 %)	84 (84.8 %)	**0.01**	73 (66.4 %)	37 (33.6 %)	**0.03**
No	2 (3.1 %)	62 (96.9 %)		26 (49.1 %)	27 (50.9 %)	
Having experience in dental injury						
Yes	17 (100 %)	0 (0 %)	**0.004**	83 (72.8 %)	31 (27.2 %)	**0.02**
No	97 (66.4 %)	49 (33.6 %)		27 (55.1 %)	22 (44.9 %)	

^a^Chi^2^ test. The bold data are statistically significant

## Discussion

The outcome of permanent tooth’s avulsion depends, among other factors, on the action taken during the first minutes after trauma [5]. The study by Unal et al. [15] showed that in 65 % of cases of tooth avulsion the time from the injury to the arrival at a dental office exceeded 60 min. The medical staff responsible for first aid in school have to be prepared to conduct immediate replantation or to preserve a tooth for delayed replantation [16]. Few studies conducted so far indicate that a gap in knowledge resulted in the inability of school nurses to handle dental trauma [11, 12]. Our findings are consistent with the data from the literature. The level of knowledge of surveyed nurses was moderate, reaching slightly above a half of the possible maximal score. Moreover, we found that most nurses were not willing to replant an avulsed tooth themselves. They indicated a dentist as the right person to provide the replantation and declared that they would wait for the parents to bring the child to a dental surgery for treatment. According to Krasner [8], there are several reasons which may influence the willingness of a school nurse to carry out an immediate replantation: the avulsion of multiple teeth causing difficulties with finding the socket a given tooth belongs to, the coexistence of more serious injuries that require immediate attention or the child being in severe pain and therefore not allowing replantation. Thus all school medical rooms should be equipped with a container and a liquid suitable for the transport of avulsed teeth to a dental surgery.

Our study did not reveal any influence of respondents’ education level on their knowledge of first aid in dental avulsion. It may result from the low percentage of subjects who declared having a higher education. The majority of Polish school nurses have only secondary or post-secondary nursing vocational education, and in our study they constituted three-quarters of the sample. Despite the fact that Poland offers the full range of higher education in nursing [17] the percentage of nurses with the Bachelor’s or Master’s degree is generally low [18]. Also, the omission of dental injuries in nursing education is observed at all levels [19]. A limited number of post-graduate training courses on such topics are offered, even though they have a very favourable influence on the ability to administer first aid to a person having a dental injury [13, 20].

Our study revealed that a strong determinant of school nurses’ knowledge was the location of the schools, and so far this aspect has not been raised. Some inequalities in the access to the healthcare for pupils from rural settings were previously observed in Poland due to the lack of a medical room in a considerable percentage of rural schools, the nurse’s care for several distant schools and frequently exceeded standards concerning the number of pupils per one school nurse [21]. Therefore we expected that nurses in rural areas would have presented a lower level of knowledge. Surprisingly, in our study, it was respondents from urban agglomeration areas who got the lowest scores. This may be explained by better access to dental clinics where the treatment of a patient with tooth injury could be provided. Such awareness may have a discouraging influence on the need to broaden the knowledge of this subject.

Our study showed that school nurses had to pursue self-education on dental trauma and we found that it was a very effective method to broaden the knowledge. It is characteristic that such individuals who encountered a tooth injury showed an increase in interest in the problem of dental injuries. Consequently, the nurses who had experience with tooth avulsion better answered the questions concerning the management of such injury. It is noteworthy that self-education concerning first aid to individuals having a tooth injury is difficult because many first-aid handbooks do not include such information within an appropriate scope [22]. Currently, few instructions and manuals have been available in the dental and medical journals [5–8]. We also confirmed the key role of the Internet as the source of knowledge. With the development of the information technology, the significance of e-health in the nursing practice is increasing [23, 24]. Distance e-learning could be especially beneficial for nurses working in rural and remote locations, saving time for travelling and letting individuals study according to their own needs and pace [25].

However, there are some inadequacies of self-education. Skills and attitudes are equally important to the knowledge in the daily practice of school nurses. Elgie at al. [26] found that online training modules were a valuable resource for improving school nurses’ knowledge and skills regarding management of emergencies, but they might not affect participants' confidence. We observed that some nurses who knew first-aid procedures in the avulsion of permanent dentition did not feel confident enough to provide a tooth replantation. Thus, the effectiveness of e-learning in dental trauma has to be carefully evaluated in future research studies. Nurses can maintain a high level of knowledge and attitudes regarding dental trauma only with a continuous help from the dental teams who should educate other medical professionals [27]. Wide education on prevention and first-aid in dental trauma should be included in public health polices [5, 27].

The response rate not exceeding 50 % should be considered as a limitation of the present study. However, in the mail survey on public nurses in Norway regarding their attitudes towards the promotion of oral health, the response rate was even lower than in our survey [28]. Probably nurses did not prioritize oral health, which may explain this relatively low response. Another limitation of the present study is that the results cannot be treated as representative for the entire country because not all school nurses in Poland work in separate offices, and some are employed by family physicians providing healthcare in the respective area. Due to the method of sample acquisition, such nurses were not included in the present study. The use of a multi-response question (‘Which medium is the best for the transport of an avulsed tooth?’) may be treated as a limitation because it could trigger difficulties with choosing a correct answer.

## Conclusion

The conducted survey revealed that the nurses’ level of knowledge of permanent tooth avulsion management was moderate and nurses working in large agglomerations presented the lowest knowledge. Due to the lack of training courses, some nurses obtained the required knowledge by self-education, mainly via the Internet. This channel maybe used to increase the public awareness of dental injuries and to deliver the evidence-based knowledge on dental trauma to non-dental medical professionals, including school nurses.
